# Extracorporeal cardiopulmonary resuscitation successfully used in a two-hour cardiac arrest caused by fulminant myocarditis: a case report

**DOI:** 10.3389/fcvm.2024.1402744

**Published:** 2024-10-22

**Authors:** Qinxue Hu, Xing Liu, Chengli Wen, Songtao Mei, Xianying Lei, Tao Xu

**Affiliations:** ^1^ Department of Critical Care Medicine, The Affiliated Hospital, Southwest Medical University, Luzhou, China; ^2^ The Third Central Clinical College, Tianjin Medical University, Tianjin, China

**Keywords:** fulminant myocarditis, cardiac arrest, CPR, ECPR, ECMO, case report

## Abstract

Fulminant myocarditis (FM) is characteristically associated with rapid progressive decline in cardiac function and high mortality, with rapid onset of hemodynamic dysfunction and severe arrhythmias. In this report, we describe a case concerning a patient clinically diagnosed with FM, marked by rapid progression leading to intractable ventricular fibrillation and subsequent cardiac arrest. Conventional cardiopulmonary resuscitation (CCPR) was performed 120 min before extracorporeal membrane oxygenation (ECMO) was initiated. This critical situation was effectively addressed through the utilization of extracorporeal cardiopulmonary resuscitation (ECPR). By providing sustained cardiopulmonary support, effective hemodynamics were obtained. Eventually, the patient made a full recovery, and discharged without neurologic complications on hospital day 13.

## Introduction

Fulminant myocarditis (FM) is a rare but serious and potentially fatal inflammatory disease of the myocardium (1, 2). The disease is characterized by an acute onset and extremely rapid progression, which is characterized by rapid deterioration over a 2-week period and can occur within 2 or 3 days (3). FM can lead to severe hemodynamic disturbances, including heart failure and arrhythmias, which are not adequately maintained properly by vasoactive medications and require mechanical circulation support (MCS) devices (4). Multi-organ failure is also common in patients with FM and can even lead to cardiac arrest (CA), usually with a poor prognosis (5, 6). Consequently, when FM is suspected or diagnosed, the treatment should be carried out in a timely manner in accordance with the “life support-based comprehensive treatment program”. Life-supportive therapy included MCS and respiratory support. MCS include ECMO, intra-aortic balloon pumping (IABP), Impella, and other cardiac assist device (7, 8).

CA caused by FM is usually associated with a high mortality rate. Clinical evidence suggests that ECMO has a beneficial effect on FM (especially in younger patients) when conventional therapies are ineffective, and venoarterial extracorporeal membrane oxygenation (VA ECMO) has been widely used in refractory CA (5). It has been reported that the duration of ECMO support ranges from 5 to 9 days and that survival to discharge is 55%–56% (9, 10). The 2019 American Heart Association guideline update states that ECPR can be used in patients with CA if traditional CPR has failed and there are trained physicians who can perform ECPR quickly (11). ECPR is a procedure where ECMO is used to enhance cardiac output and facilitate effective gas exchange, thereby maintaining organ perfusion in patients with CA when conventional cardiopulmonary resuscitation (CCPR) has failed to restore sustained return of spontaneous circulation (ROSC). ECPR can improve the prognosis by increasing perfusion to critical organs and stabilizing patients while addressing the underlying causes of the arrest (12, 13). ECPR combined with target temperature management can significantly improve the prognosis of patients with refractory CA, leading to favorable neurological outcomes (13).

## Case description

A previously healthy 26-year-old male was admitted to the intensive care unit (ICU) of our hospital with suspected FM. He had been experiencing symptoms including asthenia, adynamia, dyspnea and chest pain for at least 1 day prior to admission. Initially, he sought treatment at a local hospital, where he presented with a fever and altered mental status. Due to the severity of his symptoms, he was transferred to our hospital for advanced care. Upon examination in the emergency department, elevated cardiac markers were noted: high-sensitive cardiac troponin I was 6,240 ng/ml, myoglobin was 188.8 ng/ml, and creatine kinase was 37.89 U/L.

Upon admission, the patient exhibited several alarming symptoms: tachypnea, paleness, a weak pulse, and tachycardia. The initial examination recorded a temperature of 39°C, a respiration rate of 30 beats per minute, a pulse rate of 146 beats per minute, a blood pressure of 93/64 mmHg, and an oxygen saturation of 96%. After admission, the patient's laboratory results showed the following: electrocardiogram revealed significant ST segment elevation in leads I, II, and V1–6, high-sensitivity troponin T 4.26 ng/ml, N-terminal pro-brain natriuretic peptide 6,893 ng/ml; myoglobin 207.3 ng/ml, creatinine kinase-MB 44.47 ng/ml, aspartate aminotransferase 112.4 U/L, creatinine 131 µmol/L, uric acid 443.7 mmol/L, hemogram did not show neutrophilia, leukocytosis, or decrease in hemoglobin, but was accompanied by lymphopenia, C-reactive protein was 73.6 mg/L and procalcitonin 0.16 ng/ml. No significant abnormalities were found in the coagulation test. The admission diagnosis was FM.

At 10:33 the following day, approximately 12 h later, the patient's condition had rapidly deteriorated. Symptoms included an increased heart rate, unconsciousness, clammy skin, and a progressive drop in blood pressure. At the same time he developed ventricular fibrillation. Despite four attempts at electric defibrillation, sinus rhythm could not be restored, leading to CA and pulseless ventricular fibrillation. We immediately performed urgent intubation and started CCPR at 10:38, and communicated with the patient's relatives to perform ECMO, but due to the high costs, they could not decide whether to perform ECMO immediately. Despite repeated defibrillation efforts and 120 min of mechanical chest compressions using a LUCAS-2 device, ROSC was not achieved. At this point, the patient's blood pressure was undetectable, lactate levels exceeded 20 mmol/L. With the consent of the patient's relatives and the opinions of the multidisciplinary consultation, the ECMO team promptly established VA ECMO through peripheral cannulation of the right femoral artery with a 15-Fr arterial cannula and a 21-Fr right femoral vein at 12:10, and ECPR was successfully initiated at 12:38. To prevent distal extremity ischemia on the side of ECMO cannulation, a 6Fr arterial cannula was used to establish distal perfusion through the superficial femoral artery.

Upon initiation, the pump speed of the ECMO was set to 3,800 rpm with a flow rate of 4.0 L/min. Norepinephrine (0.8 μg/kg/min) and dobutamine (5 μg/kg/min) and were infused to maintain mean arterial pressure above 65 mmHg. Heparin was continuously infused to maintain the activated partial thromboplastin time at 1.5–2.5-fold the normal range. Regular monitoring ensured that hemoglobin levels remained above 90 g/L and platelet counts were maintained at ≥50 × 10^9^/L. Additionally, target temperature management was implemented at 35°C–36°C for 24 h.

Due to myocarditis and prolonged hypoperfusion, the patient not only experienced a loss of consciousness, registering a Glasgow Coma Score of 3, but also suffered functional impairment to the heart, kidneys, and liver, and he was experiencing anuria. To continuously remove toxins and inflammatory factors, reduce the damage caused by the inflammatory storm, and at the same time help to regulate the body fluids and acid-base balance and stabilize the internal environment, continuous renal replacement therapy was administered for additional support. Considering the echocardiographic finding of a small aortic valve opening, we introduced IABP as an adjunctive circulatory device, with an assist ratio of 1: 1 to reduce left ventricular (LV) afterload and improve myocardial perfusion. The patient's comprehensive treatment regimen included the administration of antiviral agents [acyclovir for injection, 0.5 g intravenously (iv) every 8 h over a period of 10 days], methylprednisolone sodium succinate for injection (200 mg per day for 3 days, with a gradual tapering of dosage until discontinuation on day 10), intravenous immunoglobulin (20 g IV infusion for 3 days), and antioxidants (vitamin C, 4 g per day for 1 week). Vasoactive drugs such as norepinephrine, dobutamine, and levosimendan were administered or adjusted based on blood pressure and myocardial contraction requirements. Additionally, antibiotics (piperacillin-tazobactam) and enteral nutrition support were provided throughout the treatment course.

**Figure 1:**
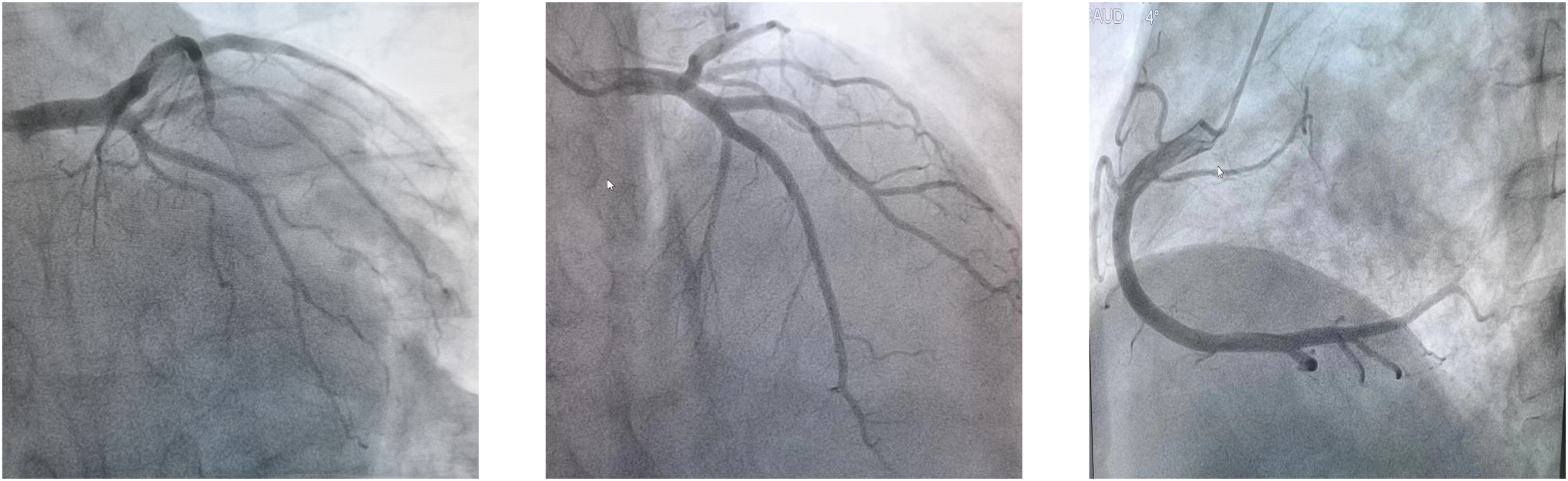
Normal function of coronary artery on coronary arteriography.

**Figure 2:**
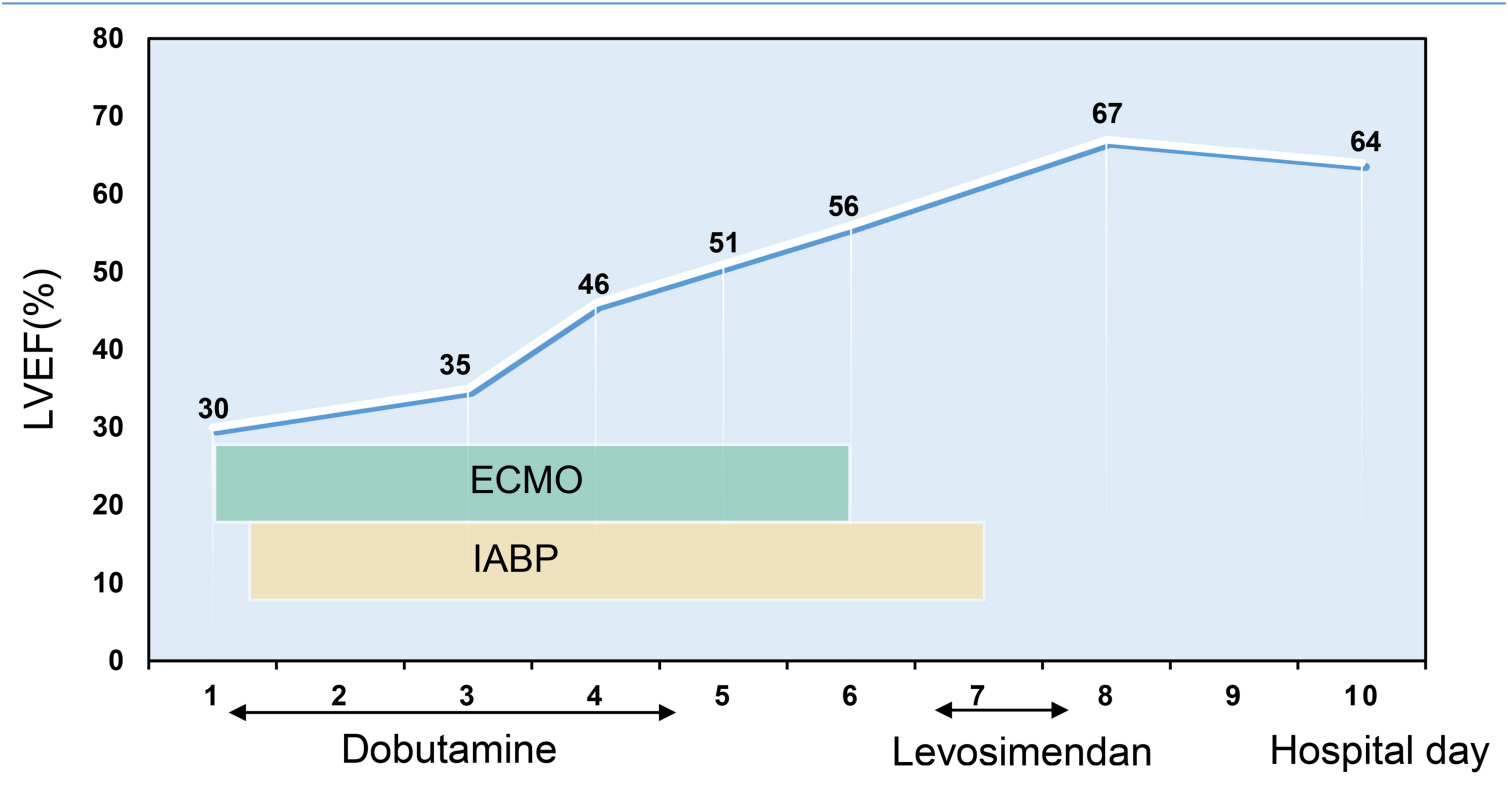
The change in patients' LVEF during the course of hospitalization.

**Figure 3:**
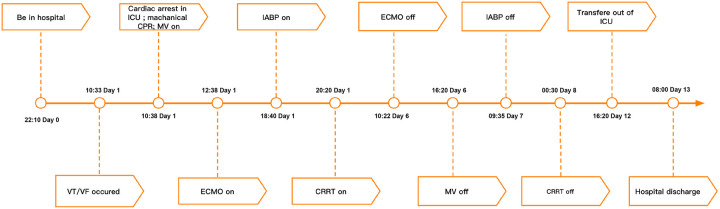
Summary of disease course and treatment flow chart. VT/VF, ventricular tachycardia/ventricular fibrillation; ICU, Intensive Care Unit; CPR, cardiopulmonary resuscitation; MV, mechanical ventilation; ECMO, extracorporeal membrane oxygenation; IABP, intra-aortic balloon pumping; CRRT, continuous renal replacement therapy.

The patient's consciousness was restored (Glasgow Coma score of 13), and sinus rhythm resumed on day 3 of ECMO support. As hemodynamics improved, we gradually discontinued vasoactive medications, leading to the patient's mean arterial pressure surpassing 65 mmHg, LV ejection fraction (LVEF) recovering from 30% to 56%, and lactate levels dropping from over 20 mmol/L to normal (Table 1). Consequently, we have gradually decreased both the ECMO flow rate and the IABP assist ratio. By day 6 of ECMO support, the patient started weaning from ECMO and mechanical ventilation; by day 7, IABP support was successfully terminated. A coronary angiography was subsequently performed, which showed no coronary artery abnormalities (Figure 1). On day 6, levosimendan (0.1 μg/kg/min) was administered to further augment left heart function upon the removal of VA ECMO. On day 12, the patient was transferred from the ICU to the cardiology department, and on day 13, he was discharged. Prior to discharge, echocardiography revealed significant improvement in LV systolic function, with an LVEF of 67% (Figure 2). Notably, the patient did not experience any ECMO-related complications throughout the entire ECMO-assisted treatment. Over the 3-month follow-up period, the patient's urine output and renal function gradually normalized post-discharge, culminating in a successful recovery without any complications. Subsequently, the patient returned to work (Figure 3).

**Table 1:** Test results throughout the clinical course.

Tests	D0	D1	D2	D3	D4	D6	D8	D9	D10	D11	D12
NT-proBNP (ng/L)	6,893	9,147	13,168	40,429	48,459	50,890	-	-	42,506	47,974	-
hs-TNT (µg/L)	4.26	7.31	6.4	7.5	7.09	4.02	-	-	0.723	-	0.178
PH	7.465	7.032	7.123	7.48	7.368	7.322	7.422	7.389	7.386	7.373	7.382
Lac (mmol/L)	1.94	>20	1.29	1.38	0.58	0.51	0.53	-	0.71	-	1.9
Urea (mmol/L)	7.49	10.3	12.59	869.1	8.33	11.21	14.25	22.16	26.38	30.23	28.3
Crea (mmol/L)	131	133	210	465.4	221	218.2	304.2	516.9	633.7	695.8	664.9
GFR (ml/min)	65.5	64.3	36.9	39.6	34.8	35.3	23.6	12.5	9.7	8.7	9.2
TBIL (µmol/L)	14.5	-	21.2	17.7	15.6	19.4	18.6	13.1	10.5	9.3	-
ALT (U/L)	23.4	-	1,256.7	869.1	729.3	482.1	247.9	163.3	116.5	86.4	-
AST (U/L)	112.4	-	2,060.5	465.4	220	93.1	31.8	30.2	21.6	17	-
CRP (mg/L)	-	73.43	75.31	69.7	42.44	6.82	25.17	8.35	8.18	-	-
PCT (ng/ml)	0.16	-	76.93	65.71	-	10.41	-	-	-	-	0.7
LVEF (%)	-	30	-	35	46	56	67	-	64	-	-
PLT (10^9^/L)	182	174	147	70	64	88	172	211	241	-	-

NT-proBNP, N-terminal pro-B-type natriuretic peptide; hs-TNT, human hypersensitive troponin T; PH, potential of hydrogen; Lac, lactate; Crea, creatinine; TBIL, total bilirubin; ALT, alanine transaminase; AST, aspartate transaminase; CRP, C-reactive protein; PCT, procalcitonin; LVEF, left ventricularinjection fraction; PLT, platelet.

## Discussion

In this case, we diagnosed him with FM, based on the symptoms of upper respiratory infection, rapid onset of circulatory failure, electrocardiographic changes, echocardiography, blood test results suggesting elevated troponin levels, and coronary artery disease was ruled out by coronary artery angiography. Our case illustrates the safe application of ECMO in patients experiencing refractory CA due to FM. To our knowledge, this constitutes a rare occurrence of an adult patient with acute FM and CA undergoing sustained CCPR for 120 min and subsequently achieving complete recovery following VA ECMO support without neurological complications. It is widely accepted that a short duration of CPR correlates with a favorable neurological outcome (14). Consequently, it is recommended that ECPR be initiated within 40 min and no later than 60 min post-onset of CA, as suggested by the Extracorporeal Life Support Organization (15). Beyond the 40-minute threshold, the survival rate with a favorable neurological prognosis significantly decreases from over 30% to approximately 15% (16, 17). Upon initiation of ECPR, extracorporeal circulation ensures perfusion to systemic organs, even in the absence of autonomic circulation. This results in enhanced coronary perfusion pressure, ROSC, and successful defibrillation compared to CCPR (18, 19).

The delay in initiating ECPR until 2 h after CCPR in this patient can be attributed to several reasons. Firstly, the patient's family expressed hesitation towards VA ECMO due to concerns regarding the high cost of treatment and uncertainty about the prognosis. Secondly, the occurrence of the patient's CA on a weekend posed a challenge, as although our ECPR team comprises well-trained ICU physicians and nurses, the unavailability of an adequately staffed on-call ECPR team throughout the day was limited. Despite the delay, the decision to proceed with ECMO was based on several crucial factors. Firstly, the patient's young age rendered him a potential candidate for improved outcomes with ECMO. Secondly, the CA occurred in the ICU, where high-quality CPR was promptly initiated, ensuring adequate blood flow to vital organs, especially the heart and brain, with zero “no-flow time.” This circumstance significantly contributed to a favorable prognosis for neurological recovery. Thirdly, the underlying condition precipitating the CA was deemed potentially reversible. Therefore, despite CPR lasting 120 min, we chose to perform ECPR on the patient, considering the aforementioned factors.

Initiating ECPR within 60 min following in-hospital CA (IHCA) is deemed potentially beneficial for both survival and neurological outcomes. Prolonged CPR duration correlates with a gradual decline in the rate of neurologically favorable survival (16). Time plays a critical role, as every additional 10 min of CPR beyond 30 min corresponds to a 25% reduction in ECPR patient survival rates (13). Research indicates that if the low-flow time exceeds 90 min before the initiation of ECPR, the survival rate drops to below 10% (13). Hence, the recommended “threshold” for commencing ECPR is within 60 min, as patients exceeding this timeframe rarely achieve favorable clinical outcomes (20). However, with advancements in national medical practices, ongoing ECMO technology innovations, enhanced medical system accessibility, and an emphasis on team collaboration, instances of successful resuscitation with low perfusion times exceeding 60 min have been documented (12, 15).

In our patient, ECMO was initiated 120 min after CPR, resulting in a complete recovery without ECMO-related complications or neurological deficits. Several factors contributed to this favorable outcome, including the timely occurrence of IHCA, enabling prompt and high-quality CPR, and aggressive post-resuscitation care. Crucially, the prompt initiation of ECMO support played a pivotal role, and it is possible that combining ECPR with TTM could further enhance neurological outcomes. Furthermore, the consistent and effective mechanical CPR delivered by the LUCAS-2 device likely played a significant role in achieving favorable neurological outcomes despite prolonged CPR duration. A previous study has shown that the duration of resuscitation before initiating ECPR with mechanical CPR did not necessarily impact survival or neurological outcomes (12). While conventional chest compressions can be effective initially, their quality of compression may deteriorate due to rescuer fatigue during prolonged CPR (21). In contrast, mechanical chest compression devices like LUCAS-2 do not suffer from fatigue and can deliver high-quality compressions consistently, ensuring continuous blood and oxygen delivery to vital organs (22). Although existing studies may not indicate the superiority of mechanical chest compression devices over traditional methods in CCPR, their integration with ECPR presents a distinct paradigm worth exploring further (12, 23, 24).

VA ECMO may induce LV overload and dilation; however, when coupled with an IABP, it proves effective in reducing LV afterload and enhancing coronary blood flow (25). The IABP is frequently used as an adjunctive device for LV unloading (26). In this case, VA ECMO provided effective cardiopulmonary support, allowing sufficient time for the recovery of cardiopulmonary function. Simultaneously, the concomitant use of IABP reduced LV afterload, resulting in the transformation of the non-pulsatile perfusion provided by VA ECMO into pulsatile perfusion, and also to facilitate myocardial recovery (26).

With an increasing number of case reports illustrating patients achieving favorable neurological outcomes with ECPR using VA ECMO despite prolonged CCPR (over 60 min) (12, 14), the decision to initiate ECMO support remains challenging. Whether the timing of the initiation of ECPR can be extended requires further validation through additional high-quality studies. CA is a sudden-onset and critical event, broadly classified as out-of-hospital CA (OHCA) and IHCA. ECPR can be implemented across various settings, including emergency ambulances, operating rooms, and hospital wards. However, it represents a time-sensitive and complex intervention necessitating well-coordinated teamwork, clearly defined roles, and a team of well-trained healthcare providers. Establishing a dedicated 24-hour ECMO team capable of promptly performing ECPR within a limited time frame is essential (27).

The successful weaning from ECMO represents a provisional and transitional approach contingent upon timely treatment of the underlying cause of cardiogenic shock or witnessing an improvement in cardiac function on its own (28). In this specific case, the patient suffered from FM, a self-limiting and reversible condition, and the CA occurred within the hospital, where prompt and effective CPR was administered. Consequently, the meticulous selection of eligible patients before ECMO applications becomes crucial for enhancing survival rates. Suitable candidates may encompass individuals with a reversible etiology for CA and those experiencing a witnessed CA with immediate administration of high-quality CPR (29).

## Conclusion

The clinical message derived from this case is very clear: ECPR emerges as a clinically feasible therapeutic option for supporting patients grappling with refractory CA. Prompt mechanical CPR can ensure the administration of high-quality chest compressions, potentially buying more time for the initiation of ECPR. Furthermore, the judicious use of an IABP at the appropriate time not only improved coronary blood flow but also effectively unloaded the LV, thereby averting complications arising from LV overload.

## Data Availability

The original contributions presented in the study are included in the article/Supplementary Material, further inquiries can be directed to the corresponding author.
